# Time to link camel genomics and traits by bridging the phenotypic gap

**DOI:** 10.3389/fgene.2025.1627229

**Published:** 2025-07-31

**Authors:** Faisal Almathen, Bashir Salim

**Affiliations:** ^1^ Camel Research Center, King Faisal University, Al-Ahsa, Saudi Arabia; ^2^ Department of Public Health, College of Veterinary Medicine, King Faisal University, Al-Ahsa, Saudi Arabia

**Keywords:** camel genomics, genotypic-phenotypic correlation, selective breeding, genetic markers, international genomic networks

## Abstract

Camels (*Camelus dromedarius* and *Camelus bactrianus*) are indispensable to the economy and culture of arid and semi-arid regions, providing milk, meat, transportation, and labor while demonstrating remarkable adaptations to extreme environments. Recent advances in camel genomics have unraveled key genetic insights related to diversity, physiological adaptation, and productivity traits. However, translating these genomic discoveries into practical applications remains limited by a critical gap in phenotypic data, standardized trait recording, and robust pedigree infrastructure essential foundations for implementing genomic selection (GS) effectively. The lack of high-density SNP arrays, variable linkage disequilibrium patterns, and incomplete genome assemblies further complicate efforts to identify causal variants, cautioning against overinterpreting GWAS results. This review provides a comprehensive analysis of camel genomics, emphasizing key genetic markers associated with growth, meat and milk production, coat color, athletic performance, environmental adaptation, cartilage integrity, and behavioral traits. Additionally, it highlights the importance of modeling genotype-by-environment interactions (G × E) and adopting advanced statistical approaches, such as random regression and reaction norm models, to capture complex trait architectures. Drawing lesson from other livestock, we propose a strategic roadmap that includes the development of high-density SNP arrays, improved genome assemblies, standardized trait recording, and establishment of large, connected training populations. International collaboration through a camel genomics consortium is essential to harmonize data, enhance genetic connectedness, and enable multi-environment evaluations. Addressing these research gaps will facilitate the development of precision breeding, climate-resilient livestock strategies, and sustainable conservation initiatives, ensuring that camels continue to thrive amid growing environmental and economic challenges.

## Graphical Abstract

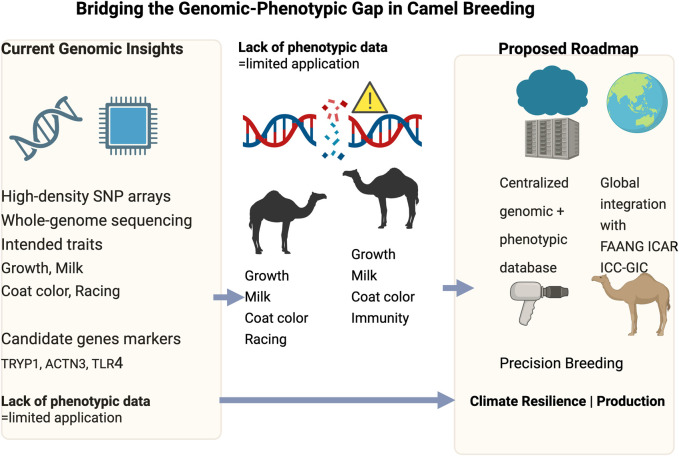

## 1 Introduction

Camels have been integral to human societies for millennia, particularly in arid and semi-arid regions where they serve as primary sources of milk, meat, leather, and wool while also functioning as reliable means of transportation and labor (Faye and Konuspayeva, 2012). The dromedary (*Camelus dromedarius*), which dominates in North Africa, the Middle East, and South Asia, and the Bactrian camel (*Camelus bactrianus*), native to Central Asia, exhibit remarkable physiological adaptations that enable survival in harsh desert climates (Burger et al., 2019). These adaptations include enhanced water metabolism, thermoregulation mechanisms, and an ability to extract nutrients from fibrous, low-quality forages, allowing camels to thrive in environments where other livestock struggle (Bouâouda et al., 2014; Wu et al., 2014).

Advancements in genomic technologies have significantly improved our understanding of the genetic basis of these adaptations, along with economically important traits such as growth, reproduction, milk yield, and disease resistance (Berry et al., 2014; Erdoğan et al., 2024; Yao et al., 2024). The sequencing of the dromedary and Bactrian camel genomes has provided valuable insights into evolutionary history, genetic diversity, and domestication events (Fitak et al., 2020). In parallel, genome-wide association studies (GWAS) and transcriptomic analyses have identified key genes influencing metabolic regulation, immune response, and reproductive efficiency, further expanding our knowledge of the genetic architecture underlying camel traits (Yao et al., 2023; Jirimutu et al., 2012).

Despite these advancements, the translation of genomic research into practical breeding applications remains hindered by a critical gap in phenotypic data collection and standardization. Unlike cattle and sheep, where genomic selection programs are well established, camels lack structured recording systems for economically relevant traits such as growth rate, milk yield, and racing performance (van der Werf, 2013; Mrode et al., 2019; Bahbahani et al., 2019). The absence of standardized phenotypic assessment protocols has resulted in fragmented and inconsistent datasets, thereby impeding the development of meaningful genotype-phenotype associations (Wu et al., 2023).

Another major challenge is the underrepresentation of camels in global livestock genomic networks, which has limited comparative studies and slowed genetic progress (Bahbahani et al., 2019). While cattle, sheep, and goats have benefited from large-scale genomic sequencing initiatives and well-established selection programs, camels remain understudied and underfunded in comparison. The lack of integration into major international livestock genomic consortia restricts access to advanced analytical tools, genomic resources, and cross-species comparative approaches that could otherwise accelerate camel genomic research and breeding advancements.

This review emphasizes the urgent need to bridge this gap by integrating genotypic and phenotypic research, developing harmonized trait measurement protocols, and capitalizing on technological advancements such as automated phenotyping systems and AI-driven data analysis (Billah et al., 2025). Additionally, we highlight the critical role of international collaborations in advancing camel genomics and breeding strategies. By providing a comprehensive assessment of the current state of camel genomic research, this review identifies key genetic markers linked to economically important traits, examines the barriers preventing genomic tools from being widely applied, and explores the potential for global research partnerships to accelerate genomic advancements. Furthermore, we propose strategic solutions for establishing standardized phenotypic trait evaluation frameworks that align with global genomic selection programs, ensuring that camel breeding and conservation efforts are data-driven, sustainable, and future-ready.

## 2 Methods

This review was conducted as a narrative literature review aimed at synthesizing current knowledge on camel genomics, with a particular focus on the integration of genotypic and phenotypic research to bridge the phenotypic gap. It is not a systematic review or a meta-analysis. The review draws from a comprehensive analysis of relevant literature published in English between 2010 and 2025. A thorough literature search was performed using databases such as PubMed, Scopus, Web of Science, and Google Scholar. The search terms included “Camel genomics,” “Genotype-phenotype correlation in camels,” “Camel breeding and genetic markers,” “Camel trait mapping,” and “Camelid genetics.” Additional studies were identified through backward citation tracking from key review articles and relevant references.

Inclusion criteria for the review comprised peer-reviewed articles published in English between 2010 and 2025, focusing on camel genomics, genetic markers, genotype-phenotype correlations, or the application of genomic tools in camel breeding. Studies that provided insights into productivity traits such as milk yield, growth rate, disease resistance, environmental adaptation, or performance traits were prioritized. Exclusion criteria included studies focusing exclusively on other livestock species without relevance to camels, articles lacking clear emphasis on genetics or genomic research in camels, and conference abstracts, editorials, or commentaries without substantial primary or secondary data. An initial screening of titles and abstracts was performed to assess relevance, followed by a full-text review of articles meeting the inclusion criteria. Any discrepancies in inclusion decisions were resolved through consensus between the authors. The final selection comprised studies that provided relevant insights into camel genomic research and its links to phenotypic traits.

Given the narrative nature of this review, no formal meta-analytic statistical methods were applied. Instead, the review synthesized qualitative insights from the selected literature to provide a comprehensive overview of key genetic markers, phenotypic traits, and research gaps. Where appropriate, summary statistics reported in the primary studies were included to support specific points (e.g., SNP counts, gene associations).

## 3 Decoding camel genomics: linking genetic markers to phenotypic traits

Recent advances in genomic research have facilitated the identification of multiple genetic markers associated with key production traits in camels, including growth rate, meat and milk production, coat color, athletic performance, disease resistance, environmental adaptation, cartilage integrity, and behavioral traits. Whole-genome sequencing has generated extensive genetic data, yet the lack of corresponding systematic phenotypic records has impeded the establishment of meaningful genotype-phenotype associations. Addressing this gap requires comprehensive phenotyping strategies and the integration of genetic insights into breeding programs to optimize productivity and resilience (Table 1).

**TABLE 1:** Decoding camel genomics a systematic framework linking genetic markers to phenotypic traits and future research needs.

Phenotypic category	Trait	Key genomic markers (Genes/SNPs) and reference	Genomic findings	Current phenotypic knowledge	Research gap
Growth & Morphometrics	Body size, movement dynamics	ACTR3B, SERAC1, TBX15, ITGA7, TRAPPC9, CIITA (Sani et al., 2022)	99 SNPs linked to growth traits; key genes ACTB, SOCS1, ARFGEF1	Limited datasets on skeletal and body measurement variations	*Correlation needed between growth-associated SNPs and actual body dimensions across age groups*
Growth & Morphometrics	Growth traits in Pakistani breeds	SNPs linked to growth at different ages in Marecha and Lassi camels (Sabahat et al., 2023)	Growth-associated genetic markers identified via GWAS	Growth trajectories recorded, but genetic linkage remains unexplored	*Longitudinal studies needed to track genetic impact on phenotypic growth variation*
Meat Production	Muscle mass, meat yield	MSTN (Bruno et al., 2022)	MSTN variants impact muscle function and growth regulation	Meat quality traits (marbling, tenderness) largely uncharacterized	*Need to link MSTN variants with carcass yield and meat composition*
Milk Production	Lactation traits, milk yield	TYRP1, DLC1, GPC5, SLC24A4, NEMP2, SLC14A1 (Alsubaie et al., 2024)	28 SNPs linked to milk yield and composition	Dairy performance data exists but lacks genetic correlation studies	*Genotype-phenotype studies needed for improving dairy selection*
Milk Production	Mammary gland development	PRKAB2, PRKAG3, PLCB4, BTRC, GLI1, WIF1, DKK2, FZD3, WNT4 (Yao et al., 2023)	2,851 differentially expressed genes related to mammary gland biology	Molecular mechanisms of lactation poorly studied in camels	*Needs functional validation linking mammary genes to milk yield traits*
Coat Color Variation	White vs. black pigmentation	ANKRD26, GNB1, TSPYL4, TEKT5, SNAI1, TBX15, COL4A4, CIITA (Sani et al., 2022)	SNAI1 interacts with MC1R, ASIP, KIT to regulate melanin biosynthesis	Visual classifications of coat color exist, but genetic basis remains underexplored	*More large-scale population studies integrating genetics and coat color variation*
Athletic Performance	Racing ability, endurance	ACTN3, MSTN, PPARGC1A, CKM, VEGFA (Bahbahani et al., 2025)	Genes linked to muscle metabolism and endurance	Performance data exists, but lacks genetic linkage to speed, stamina	*Genomic selection studies needed for elite racing lines*
Environmental Adaptation	Metabolism, thermoregulation	TRNAI-AAU, BAG5, SEPTIN7, SLC13A1, PCED1B, BMPR1B, JAKMIP2, NOTCH2 (Almathen et al., 2024)	Genomic signatures of adaptation in desert environments	Physiological data on heat stress adaptation exists, but lacks genetic validation	*GWAS studies linking genetic markers to heat tolerance traits needed*
Environmental Adaptation	Selection signatures in Iranian camels	FUT1, SLC9A3, SLC9A2, SLC26A6, SLC26A3 (Khalkhali-Evrigh et al., 2022)	Genes linked to osmoregulation and immune response under selection	Phenotypic adaptation observed, but no direct genotype-phenotype studies	*Needs functional studies validating selection effects on phenotype*
Disease Resistance	MERS-CoV susceptibility	MHC class I (HLA-A-24-like), MHC class II (HLA-DPB1-like), PTPN4, MAGOHB, DNAH7 (Lado et al., 2021)	Immune gene variants associated with virus replication	Serological studies available but lack genetic correlations	*Need to establish genetic risk factors for MERS-CoV susceptibility*
Disease Resistance	CCHFV susceptibility	FCAR, CLEC2B (Lado et al., 2021)	FCAR and CLEC2B linked to CCHFV resistance	Phenotypic resistance observed but lacks genomic validation	*Functional studies needed to confirm immune response mechanisms*
Cartilage & Energy Balance	Skeletal integrity, metabolism	ATF7IP2, NAA16, MTRF1, DLG1, UBA52, TMEM59L, KLHL26, CRLF1, CRTC1, WDR7 (Bahbahani et al., 2019)	Genes linked to cartilage formation and metabolic homeostasis	Bone structure and metabolic efficiency poorly characterized	*Functional genomics studies needed linking genes to performance traits*
Behavior & Biomechanics	Body movement, sensory functions	POU2F2, ZNF574, GRIK5, PCDH15, NFASC, SAMD12, SPAG16 (Iglesias Pastrana et al., 2023)	16 markers associated with body measurements, biomechanics, and behavior	Behavioral traits lack genetic evaluation in camel studies	*More research needed linking genetics to temperament and gait*

### 3.1 Growth traits

Studies have identified significant genetic variants associated with growth traits such as birth weight, daily gain, and body weight. A total of 99 single nucleotide polymorphisms (SNPs) have been mapped to 22 genes involved in biological functions such as calcium ion binding, protein kinase activity, and cytokine signaling (Bahbahani et al., 2019). Notable candidate genes such as *EFCAB5*, *MTIF2*, *MYO3A*, *TBX15*, *IFNL3*, *PREX1*, and *TMOD3* have been linked to muscle development, metabolism, and immune function (Sani et al., 2022). These genes are essential in regulating growth pathways, with *TBX15* playing a crucial role in skeletal development and *MYO3A* involved in myosin function, which influences muscle contraction and structure. Understanding the functional implications of these genes can provide a genetic basis for selecting camels with superior growth performance, but validation requires extensive phenotypic characterization.

### 3.2 Milk production

Milk production is a key economic trait in camels, yet the genetic basis of lactation traits remains poorly studied. Recent genomic studies have identified genetic markers within *TYRP1, DLC1, GPC5, SLC24A4, NEMP2,* and *SLC14A1* that are associated with milk yield and composition (Alsubaie et al., 2024). The TYRP1 plays a crucial role in mammary gland development, while GPC5 is linked to lipid metabolism. Despite these findings, genotype-phenotype correlation studies remain scarce, limiting the application of genomic selection for dairy improvement. Additionally, mammary gland development is regulated by *PRKAB2, PRKAG3, PLCB4, BTRC, GLI1, WIF1, DKK2, FZD3,* and *WNT4*, with over 2,851 differentially expressed genes identified in camel lactation studies (Yao et al., 2023). However, the molecular mechanisms of lactation remain poorly characterized, necessitating functional validation of candidate genes to enhance milk production efficiency. Whole-genome resequencing has also revealed 13 SNPs linked to milk yield and 18 SNPs influencing milk composition, particularly fat and protein content (Yao et al., 2024). Notably, *NR4A1, ADCY8, ROR2, NRG3, IGF1R, RHOA, PCSK9, CRKL, LOC105075649, CSN2*, and *CSN3* have been implicated in these traits. High-yielding camels exhibit lower fat and protein concentrations, suggesting a trade-off between yield and milk quality. Nevertheless, large-scale functional validation of SNPs and genomic association studies across diverse camel populations are essential to refine breeding strategies for enhanced dairy productivity (Table 1).

The absence of standardized phenotyping protocols remains a major barrier to effective genomic selection in camels. Implementing automated lactation monitoring systems, real-time milk composition analysis, and multi-omics approaches will be crucial to bridging the genotype-phenotype gap and improving genetic selection for dairy traits. Future research should prioritize genome-wide association studies (GWAS) to identify and weight causal variants for breeding programs. Although epigenetic profiling holds promise for understanding gene regulation and trait expression, its routine application in camel breeding remains limited. Therefore, initial efforts should focus on validating candidate variants identified by GWAS, while laying the groundwork for integrating epigenetic insights into long-term genomic selection strategies.

### 3.3 Coat color

Coat colour is an important phenotypic trait in camels, often linked to breeders’ preference, thermoregulation, and adaptation. A total of 9 SNPs have been identified for white coat color and 13 SNPs for black coat color, with *SNAI1, MC1R, ASIP,* and *KIT* playing essential roles in melanin biosynthesis and pigmentation (Sani et al., 2022). SNAI1 interacts with MC1R and ASIP to regulate pigmentation pathways, suggesting that selective breeding based on coat color could be informed by genomic selection. However, phenotypic expression of coat color is also influenced by epigenetic regulation and environmental factors, requiring further studies to dissect the genetic and environmental interactions that define pigmentation patterns in camels (Barazandeh et al., 2019).

### 3.4 Athletic performance

Athletic performance is a significant trait in racing camels, particularly in the Arabian Peninsula, where camel racing is a cultural and economic enterprise. Genomic studies have identified key markers associated with endurance, muscle efficiency, and metabolic adaptation. Genes such as *ACTN3, PPARGC1A, CKM,* and *VEGFA* have been implicated in muscle metabolism, oxygen transport, and ATP generation, all of which influence speed and stamina (Bahbahani et al., 2025). *ACTN3* is particularly relevant, as it is a known sprint gene influencing muscle fiber composition in multiple species, including humans and horses. Similarly, VEGFA plays a crucial role in vascularization and oxygen delivery to muscles, making it a key determinant of endurance (Wagner, 2011). Despite these findings, validating these markers with real-world racing data remains a challenge due to environmental influences, training regimes, and management practices.

### 3.5 Cartilage energy balance and adaptation

Maintaining skeletal integrity and metabolic homeostasis is crucial for livestock performance, yet the genetic mechanisms underlying these traits remain poorly characterized. Recent studies, such as those by Bahbahani et al. (2019), have identified key genes (*ATF7IP2, NAA16, MTRF1, DLG1, UBA52, TMEM59L, KLHL26, CRLF1, CRTC1,* and *WDR7*) linked to cartilage formation and metabolic regulation. However, the functional roles of these genes in bone structure and energy efficiency require further exploration. Limited understanding of gene-environment interactions hampers the development of marker-assisted selection for skeletal and metabolic traits. Future research should integrate functional genomics, GWAS, and transcriptomics to establish genotype-phenotype correlations and enhance breeding strategies aimed at improving skeletal resilience and metabolic efficiency in camels.

### 3.6 Disease tolerance

Camels exhibit remarkable disease resistance, yet the genetic mechanisms underlying their immune resilience remain poorly defined. While studies have linked MHC class I (*HLA-A-24-like*), MHC class II (*HLA-DPB1-like*), *PTPN4, MAGOHB*, and *DNAH7* to MERS-CoV susceptibility (Lado et al., 2021) and *FCAR* and *CLEC2B* to resistance against Crimean–Congo hemorrhagic fever CCHFV (Lado et al., 2021), genotype-phenotype correlations remain largely unexplored. Camels’ immune adaptations, including unique single-domain antibodies (VHH/nanobodies), enhanced pathogen recognition, and robust inflammatory responses (Hamers-Casterman et al., 1993; Plasil et al., 2019; Hussen and Schuberth, 2021), suggest strong genetic determinants of disease resilience. Key immune genes *TLR2, TLR4, IFNG, IL6,* and *CD209* influence pathogen response and infection susceptibility, presenting promising targets for genomic selection. However, despite well-documented phenotypic resistance, most studies lack functional validation and integrated genomic analysis. Future research must prioritize GWAS and multi-omics approaches to establish robust genotype-phenotype associations, enabling precision breeding, targeted vaccination strategies, and improved zoonotic disease control within the One Health framework.

### 3.7 Behavior, biomechanics and environmental adaptation

The genetic basis of behavior, biomechanics, and environmental adaptation plays a crucial role in optimizing camel performance, welfare, and resilience to extreme climates. Recent genomic studies have identified key genes associated with body movement, sensory functions, and biomechanics, such as *POU2F2, ZNF574, GRIK5, PCDH15, NFASC, SAMD12,* and *SPAG16* (Iglesias Pastrana et al., 2024). Furthermore, 16 genetic markers linked to body measurements and behavioral traits provide initial molecular insights into temperament and gait. However, behavioral genetics in camels remains largely unexplored, limiting its application in genomic selection for locomotion and adaptability. Future research should integrate genetic, biomechanical, and neurophysiological approaches to establish robust genotype-phenotype associations.

Similarly, understanding the genetic basis of environmental adaptation is essential for improving camel resilience to extreme desert climates. Recent findings have identified genes involved in metabolism and thermoregulation, including *TRNAI-AAU, BAG5, SEPTIN7, SLC13A1, PCED1B, BMPR1B, JAKMIP2,* and *NOTCH2* (Almathen, 2024). These genes exhibit genomic signatures of adaptation in arid environments, though their functional roles in heat stress responses remain understudied. While physiological studies on heat tolerance exist, they lack direct genetic validation, underscoring the need for genome-wide association studies (GWAS) to establish links between genetic markers and heat tolerance traits. Additionally, research on Iranian camels has identified selection signatures in genes associated with osmoregulation and immune response, such as *FUT1, SLC9A3, SLC9A2, SLC26A6,* and *SLC26A3* (Khalkhali-Evrigh et al., 2022). These genes contribute to water and electrolyte balance, essential for survival in desert conditions. However, direct genotype-phenotype association studies are still lacking. Future functional genomics research is needed to validate selection signals, ensuring a deeper understanding of camel adaptability and informing selective breeding strategies for enhanced climate resilience.

### 3.8 Genomic selection in camel research

Currently, genomic selection (GS) in camels is in its infancy due to several challenges, including small and fragmented training populations, weak pedigree structures, and a lack of standardized trait recording systems. For GS to be effective, there must be sufficient genetic connectedness across populations, well-recorded pedigrees, and comprehensive trait data. The absence of these foundational elements represents a major barrier to the implementation of GS in camel breeding. Future efforts should focus on establishing large training populations, integrating genomic data with reliable phenotypic records, and harmonizing data collection protocols across breeding programs to facilitate accurate genomic prediction.

### 3.9 Linkage disequilibrium and GWAS interpretation

It is important to note that GWAS-derived candidate genes should be interpreted with caution, as many significant associations may reflect linkage disequilibrium (LD) with nearby loci rather than direct causation. Given the variable and often short-range LD patterns observed in camel populations, particularly in the absence of high-density SNP arrays, there is a risk of overestimating the biological relevance of associated markers. Future studies should prioritize fine-mapping approaches and functional validation to strengthen causal inferences and enhance the utility of GWAS findings.

### 3.10 Statistical models and trait architecture

Traits such as longitudinal growth, reproductive efficiency, and disease resilience are often complex and may involve significant interactions with environmental variables. Advanced statistical models, including random regression models for growth trajectories, reaction norm models for genotype-by-environment interactions, and survival analyses for disease resistance, are essential for accurate genetic evaluations. However, such models have not yet been widely implemented in camel breeding due to data limitations. Integrating these models into camel genomic studies is crucial for developing robust and reliable breeding value estimates.

### 3.11 Genomic resources–SNP arrays and genome assemblies

Despite recent advances, camel genomic resources remain underdeveloped. Available SNP arrays lack high marker density and are often derived from limited populations, restricting their utility in diverse breeding programs. Reference genome assemblies exist for both dromedary and Bactrian camels; however, their contiguity and annotation quality remain variable. Further efforts are needed to improve assembly quality through long-read sequencing technologies and pan-genome construction. The development of camel-specific imputation panels and high-density SNP arrays will be instrumental in advancing genomic studies and selection programs.

### 3.12 Pedigree and data infrastructure

A major challenge for genomic selection and AI-based breeding tools in camels is the absence of standardized pedigree recording and animal identification systems. Many camel herds rely on informal naming systems without unique IDs, leading to fragmented and unreliable pedigree data. Implementing robust, standardized animal ID and trait recording frameworks is a foundational step toward building comprehensive training populations and facilitating accurate genetic evaluations.

### 3.13 Genotype-by-environment interaction (G × E)

Given the wide geographic and climatic range of camel production systems, genotype-by-environment (G × E) interactions are highly relevant. G × E can lead to genotype re-ranking across different environments, affecting the consistency of breeding values. Reaction norm models, which allow breeding values to vary across environmental gradients, are valuable tools for capturing G × E effects and improving selection accuracy. Implementing such models will be critical for developing resilient and high-performing camel genotypes across diverse environments.

## 4 International networks supporting camel research and development

In recent years, the landscape of camel science has been bolstered by the emergence of dedicated international networks that address various facets of camel production, cultural heritage, and genetic advancement. These organisations, though varied in scope, collectively contribute to a more coordinated and strategic approach to camel research, conservation, and industry development (see Table 2).

**TABLE 2:** The international camel networks: current roles.

Camel networks website	Focus	Activities
The International Society of Camelid Research and Development (ISOCARD) https://isocard.info/	Advancing scientific research, education, and sustainable practices for camelids to improve their health, welfare, and economic value globally through international collaboration.	Organizing triennial International Camelid Conferences to foster networking among scientists
International Camel Organization (ICO) https://ico.org.sa/	Preserving camel heritage and promoting global camel-related cultural, economic, and scientific development through international cooperation.	Organizing camel festivals, establishing regional associations
World Camelids Sport (formerly International Camel Racing Federation - ICRF) https://the-icrf.net/	Promote and develop camel racing as a global sport while preserving its cultural heritage and expanding its international presence.	Organizing international camel racing events, developing unified rules and regulations
The International Camel Consortium for Genetic Improvement and Conservation (ICC-GIC) https://icc-gic.weebly.com/	Promoting development and implementing tools for genetic improvement, including identification and recording systems, genome mapping, SNP-Chip development, and full genome sequencing.	Coordinate global efforts in advancing camel genetic improvement and conserving biodiversity through research, genome mapping, bio-banking, and capacity building.

The International Society of Camelid Research and Development (ISOCARD) has become the primary scholarly platform for camelid researchers worldwide. Through its conferences, educational efforts, and collaborative research promotion, ISOCARD plays a pivotal role in fostering academic exchange and advancing applied science in camelid health, production, and genetics. Its growing influence presents an opportunity to embed phenotypic data standardization and genomic literacy across institutions in camel-producing countries.

The International Camel Organization (ICO), while rooted in cultural preservation and regional cooperation, serves as a critical enabler for camel-related science policy and institutional alignment. By engaging with both governmental bodies and private stakeholders, the ICO offers a bridge between traditional camel heritage and modern research priorities, including support for breed documentation, herd performance recording, and education on sustainable genetic practices.

World Camelids Sport, previously the International Camel Racing Federation (ICRF), governs the expanding domain of camel racing and related sports. Though not a scientific body *per se*, its structured organization of competitive events and regulatory frameworks presents a unique opportunity for introducing standardized recording of physical performance traits. These data, if collected consistently, could be invaluable for understanding the genetic underpinnings of athleticism, stamina, and metabolic efficiency in racing camels.

The International Camel Consortium for Genetic Improvement and Conservation (ICC-GIC) represents one of the most targeted efforts toward organizing camel genomic and breeding research under a unified global strategy. It promotes initiatives such as the development of high-throughput genotyping tools, biobanking for long-term genetic preservation, and the design of comprehensive animal recording systems. ICC-GIC’s collaborative model could play a key role in aligning fragmented national breeding programs and creating synergies with global livestock genomics platforms.

Collectively, these international networks are instrumental not only in advocating for camel research but also in facilitating its operationalization through policy influence, data infrastructure, and institutional capacity building. Their integration into the broader livestock genomics ecosystem, particularly through partnerships with networks such as ICAR, ASGGN, and IMGS, will be essential to advancing evidence-based camel breeding, conservation, and productivity enhancement under both local and global agendas.

## 5 The role of international genomics networks in advancing camel research

Despite significant progress in camel genomics, especially in genome sequencing and SNP discovery, the integration of standardized phenotypic data and applied breeding frameworks remains underdeveloped. To address this gap, engaging with existing international livestock genomics networks offers a practical and strategic pathway forward. Table 3 summarizes key organizations (*ICAR, ASGGN, IMGS, IMGC, ISAG, FAANG,* and *ICC-GIC*) and their potential roles in supporting camel-specific research and development.

**TABLE 3:** The role of international genomics networks in advancing camel research: current gaps and future prospects.

Society/Consortium website	Focus	Activities	Camel-specific research needs	Potential camel research initiatives	Camel-specific applications
International Society for Animal Genetics (ISAG) https://www.faang.org/	Molecular genetics in animals	Hosts conferences, comparison tests, publishes Animal Genetics	No dedicated camel genetics research group under ISAG	Establish an ISAG Camel Genetics Committee	Encourage camel parentage verification & breed identification
Functional Annotation of Animal Genomes (FAANG) https://www.faang.org/	Functional genome annotation	Generates & analyzes epigenomic data	Lack of camel FAANG data, no standardized annotation	Develop a Camel-FAANG project to map regulatory elements	Apply FAANG annotation methods to camel genomes
Livestock Phenomics Consortium	Phenomics integration	Uses imaging, biomarkers for livestock traits	No systematic camel phenomics projects	Develop a Camel Phenomics Network to track adaptation, productivity	Adapt cattle & sheep phenomics techniques for camel breeding
CGIAR Research Program on Livestock – Livestock Genetics https://livestock.cgiar.org/index.html	Improving livestock genetics for sustainable agriculture	Conducts genetic improvement research in developing countries	Camels underrepresented in CGIAR livestock programs	Initiate a Camel Sustainable Breeding Program for climate resilience	Use CGIAR livestock breeding frameworks for camels
International Committee for Animal Recording (ICAR) https://www.icar.org/	Ensure accuracy and comparability in livestock data recording systems globally	Establishes and maintains international guidelines and standards for animal identification, performance recording, milk recording, and genetic evaluation	Lack of standardized performance and phenotypic recording protocols for camel populations	Develop standardized camel performance recording systems (e.g., milk yield, fertility, growth), aligned with ICAR guidelines	Implement ICAR-compliant protocols for milk yield testing, reproduction traits, and parentage control in camels
Animal Selection, Genetics & Genomics Network (ASGGN) https://www.asggn.org/	Promote genetic improvement in livestock through genomics, especially in low- and middle-income countries	Facilitates training, capacity building, and collaboration in genomic selection and breeding programs	Limited genomic selection frameworks for camel traits in LMICs; underrepresentation in global breeding efforts	Develop capacity-building programs for genomic data collection and analysis in camels across Asian and African regions	Establish genomic selection pipelines for camels focused on production, reproduction, and disease resistance in smallholder systems
International Mammalian Genome Society (IMGS) https://www.imgs.org/	Advance the study of mammalian genomes to understand genome structure, function, and evolution	Hosts international conferences, promotes genome annotation, comparative genomics, and bioinformatics resources	Need for refined genome annotation and functional studies in camels; limited inclusion in mammalian genome reference databases	Engage camel genomics researchers in IMGS workshops and annotation jamborees; integrate camel data into comparative genomics platforms	Improve reference genome assemblies for dromedaries and Bactrian camels; identify functional elements linked to adaptation and disease resistance
The International Milk Genomics Consortium (IMGC) https://www.milkgenomics.org/	Promote understanding of milk composition and its impact on health through genomics and systems biology	Hosts symposia, supports milk composition studies, omics research, and data sharing	Limited genomic and proteomic profiling of camel milk; need to characterize bioactive components unique to camel milk	Inclusion of camel milk in IMGC multi-species studies; omics-based profiling of camel milk traits	Improve understanding of camel milk’s nutritional and therapeutic value; identify genetic markers linked to milk yield and quality traits

The International Committee for Animal Recording (ICAR) has long established global protocols for livestock identification, performance recording, milk testing, and genetic evaluation. However, these standards have yet to be widely applied to camels. Integrating ICAR-compliant recording systems for milk yield, reproductive traits, growth performance, and parentage control in camels would create harmonized datasets critical for launching genomic selection programs and improving breeding accuracy.

The Animal Selection, Genetics & Genomics Network (ASGGN) focuses on capacity building and genomic infrastructure development in low- and middle-income countries (LMICs). Given that camel production is often managed by smallholders and pastoralist communities in LMICs, ASGGN represents a natural partner for initiating camel-specific training, phenotypic data collection systems, and genomic selection pipelines targeting local adaptive traits and production efficiency.

The International Mammalian Genome Society (IMGS) promotes functional genome annotation and comparative genomics across mammalian species. Although camel genomes have been sequenced, annotation remains incomplete. Involvement in IMGS-led genome curation and comparative analysis efforts would enhance the resolution of camel genome resources and allow exploration of unique adaptations to arid environments, metabolism, and disease resistance.

The International Milk Genomics Consortium (IMGC) applies multi-omics approaches to dissect milk composition, functionality, and health impacts in humans and livestock. Camel milk, known for its therapeutic and hypoallergenic properties, has not been included in these comparative datasets. Integrating camel milk into IMGC studies could help identify key bioactive compounds and genetic loci underlying milk quality traits—information valuable for both breeding and functional food development.

The International Society for Animal Genetics (ISAG) provides global leadership in the development of DNA-based tools for parentage verification, breed identification, and genetic diversity studies. Although ISAG has not yet formally established a camel-focused working group, initiating such a platform could drive standardization of genotyping methods and facilitate international comparison of camel genetic resources.

The Functional Annotation of Animal Genomes (FAANG) consortium supports the generation of comprehensive functional genomic datasets to interpret genome function across tissues and species (Harrison et al., 2021). Camels are currently absent from FAANG initiatives. Launching a Camel-FAANG subproject would enable transcriptomic, epigenetic, and regulatory element annotation, which is vital for translating sequence data into actionable biological insight.

Finally, the International Camel Consortium for Genetic Improvement and Conservation (ICC-GIC), though still emerging, has the potential to unify global efforts in camel breeding and conservation genomics. It can serve as a coordinating body to align research agendas, share genomic resources, and represent camelid interests within broader livestock genomics frameworks.

Taken together, the initiatives summarized in Table 3 reveal multiple points of entry for camels into global livestock genomics platforms. Strategic alignment with these networks would not only elevate the visibility of camel research but also help bridge the widening gap between rapidly advancing genomic resources and the underdeveloped phenotypic infrastructure. Mobilizing these collaborations is essential for realizing precision breeding, sustainability, and resilience in camel production systems under climate change and evolving market demands.

## 6 Recommendations and future directions

To bridge the gap between camel genomics and practical breeding applications, we propose the following recommendations:

• Short-term priorities should focus on standardizing trait recording systems, building reliable pedigree databases, and improving the quality of genome assemblies to support foundational genetic research.
• Long-term goals should include establishing large, genetically connected training populations and implementing genomic selection programs tailored to camel production systems in different regions.
• International collaboration is crucial. We recommend establishing a camel genomics consortium, modeled after MACE in cattle, to harmonize data, connect populations, and facilitate multi-environment evaluations.
• Methodological considerations include caution against overinterpreting GWAS results, the importance of modeling G × E interactions and accounting for LD structure, and the need to apply robust statistical models that can handle the complexity of camel trait architectures.

## 7 Conclusion and future perspectives

The integration of genomic and phenotypic research in camels is crucial for advancing genetic improvement, productivity, and resilience in arid and semi-arid environments. While recent genomic studies have identified key markers linked to milk yield, disease resistance, and environmental adaptation, the lack of standardized and comprehensive phenotypic data continues to constrain the implementation of genomic selection programs. Addressing key gaps such as LD structure, SNP array development, pedigree recording, and robust statistical modeling coupled with international collaboration and data sharing will enable the development of reliable genomic selection tools. Ultimately, a coordinated effort to bridge the genomic-phenotypic divide will transform camel breeding into a data-driven enterprise, essential for securing food systems and sustaining camel-based livelihoods in vulnerable regions.
